# Encapsulation and Evolution of Polyynes Inside Single-Walled Carbon Nanotubes

**DOI:** 10.3390/nano14110966

**Published:** 2024-06-02

**Authors:** Kunpeng Tang, Yinong Li, Yingzhi Chen, Weili Cui, Zhiwei Lin, Yifan Zhang, Lei Shi

**Affiliations:** 1 State Key Laboratory of Optoelectronic Materials and Technologies, Guangdong Basic Research Center of Excellence for Functional Molecular Engineering, Nanotechnology Research Center, School of Materials Science and Engineering, Sun Yat-Sen University, Guangzhou 510275, China; 2 South China Advanced Institute for Soft Matter Science and Technology, School of Emergent Soft Matter, South China University of Technology, Guangzhou 510640, Chinazhiweilin@scut.edu.cn (Z.L.); 3 Huzhou Key Laboratory of Environmental Functional Materials and Pollution Control, School of Engineering, Huzhou University, Huzhou 313000, China

**Keywords:** polyynes, linear carbon chains, (6,5) single-walled carbon nanotubes, Raman spectroscopy

## Abstract

Polyyne is an sp-hybridized linear carbon chain (LCC) with alternating single and triple carbon–carbon bonds. Polyyne is very reactive; thus, its structure can be easily damaged through a cross-linking reaction between the molecules. The longer the polyyne is, the more unstable it becomes. Therefore, it is difficult to directly synthesize long polyynes in a solvent. The encapsulation of polyynes inside carbon nanotubes not only stabilizes the molecules to avoid cross-linking reactions, but also allows a restriction reaction to occur solely at the ends of the polyynes, resulting in long LCCs. Here, by controlling the diameter of single-walled carbon nanotubes (SWCNTs), polyynes were filled with high yield below room temperature. Subsequent annealing of the filled samples promoted the reaction between the polyynes, leading to the formation of long LCCs. More importantly, single chiral (6,5) SWCNTs with high purity were used for the successful encapsulation of polyynes for the first time, and LCCs were synthesized by coalescing the polyynes in the (6,5) SWCNTs. This method holds promise for further exploration of the synthesis of property-tailored LCCs through encapsulation inside different chiral SWCNTs.

## 1. Introduction

Carbyne, an sp-hybridized linear carbon chain (LCC) with properties independent of its length, has been predicted by first-principle calculations to be the stiffest material. Moreover, it possesses superior electrical and thermal properties compared to sp^2^-hybridized carbon nanotubes and graphene [1]. Polyyne is an sp-hybridized LCC with alternative single and triple carbon–carbon bonds, which is very reactive due to its one-dimensional structure [2]. Short polyyne can be stabilized by using hydrogen as terminations on both ends [3,4,5,6]. Larger chemical ending groups should be applied for longer polyynes to hinder their reactivity [7,8,9,10]. However, cross-linking reactions between the polyyne molecules are unavoidable, making it difficult to synthesize long polyynes. The longest polyynes consisting of from 44 and 48 to recent 68 and 120 carbon atoms have been synthesized using different ending groups and synthesis strategies (e.g., Glaser coupling) [11,12,13,14]. However, the properties, e.g., optical absorption, of the long polyyne did not become saturated, suggesting that the synthesized longest polyyne is still not long enough to achieve carbyne, an infinite LCC, or a long LCC with its property independent of its length [3,15,16,17]. When a chain is longer than the longest polyyne synthesized by organic synthesis in a solvent, the chain is called an LCC instead of polyyne for clarification, especially carbon chains inside carbon nanotubes (CNTs), as introduced below.

Another strategy to stabilize long polyynes is using CNTs as a nanoreactor for synthesis, and the encapsulated LCCs can be protected by the CNTs. Thus, the synthesis of long LCCs inside CNTs is achievable. Indeed, a long LCC consisting of more than 100 carbon atoms was prepared inside multi-walled CNTs (MWCNTs) by the arc-discharge method, which is longer than the longest free-standing polyyne [18]. The LCCs was formed simultaneously with the MWCNT; thus, the length distribution of the LCCs is not controllable. In 2015, Andrade et al. utilized scanning transmission electron microscopy coupled with electron energy loss spectroscopy to examine the cross-sectional structure of an LCC@MWCNT, conclusively verifying the presence of a 1D LCC configuration inside the innermost tubes of an MWCNT [19]. Recently, double-walled CNTs (DWCNTs) as starting materials were post-annealed at high temperatures, and long LCCs were formed inside the DWCNTs by reconstructing the structure of the nanotubes [20]. The LCCs were composed of tens to thousands of carbon atoms, whose length can be roughly tailored by the diameter of the DWCNTs. More importantly, the properties of the long LCC inside the DWCNT are independent of its length but are determined by the characteristics of the DWCNT itself [21]. Therefore, the long LCC can be recognized as carbyne according to its definition. To go a step further, SWCNTs with only one wall are able to avoid the influence on the LCCs from the outer walls through the inner walls of the DWCNTs, thus allowing the properties of the LCCs to be precisely tailored. In addition, highly pure single-chiral SWCNTs are available for the confined synthesis of the LCCs with well-defined properties while the DWCNTs with a broad diameter distribution synthesize various LCCs with varied properties. However, the preparation of long LCCs inside SWCNTs is challenging due to their lower thermal stability compared to DWCNTs.

Recently, a route for the preparation of LCCs inside SWCNTs combined the chemical synthesis and the confined synthesis inside CNTs. Chemical reaction inside CNTs is a promising technique to grow new nanomaterials in general [22,23,24,25]. In principle, short polyynes inside the same CNTs could be moved freely along the nanotube, allowing the polyynes to meet each other and fuse into a long LCC. Polyyne molecules (C_10_H_2_) consisting of 10 carbon atoms were filled inside DWCNTs at 80 °C, and then long LCCs were synthesized by coalescing the C_10_H_2_ via high-temperature annealing [26]. Similarly, polyynes with 8–14 carbon atoms were filled inside SWCNTs at 80 °C under high pressure and then fused into long LCCs via high-temperature annealing [27]. The instability of polyynes made them decompose during the filling process in both cases. This decomposition reduced the filling ratio of polyynes, which, in turn, resulted in a low yield of LCCs inside the CNTs.

Here, to increase the filling ratio of polyynes inside SWCNTs, SWCNTs with an average diameter of around 0.9 nm were utilized as nanoreactors, which is larger than the SWCNTs previously used in other studies. In addition, the filling was performed at a low temperature to avoid any decomposition of the polyynes. As a result, signals of filled polyynes were clearly observed by Raman spectroscopy without using surface-enhanced Raman spectroscopy (SERS), suggesting the high filling ratio of polyynes inside SWCNTs. Remarkably, when the polyynes were filled inside single chiral (6,5) SWCNTs, the Raman signals of polyynes were even stronger than the G-band of the SWCNTs, illustrating an ultra-high filling ratio of polyynes. Subsequent annealing at high temperatures for the filled samples resulted in a coalescence between the polyynes. Thus, LCCs were formed inside the SWCNTs. Interestingly, some Raman signals of the obtained LCCs were first observed, which may belong to the LCCs with intermediate lengths in the range of 10–30 nm. We believe that this synthesis route not only offers insights into the growth mechanism of the LCC but also opens up possibilities for the production of the LCC with designed properties in the future.

## 2. Materials and Methods

### 2.1. Sample Preparation 

Polyynes (C_2n_H_2_, n is a positive integer, n = 4–8) were prepared via the submerged arc-discharge method [28]. Two graphite electrodes with a diameter of 6 mm were fixed into a four-mouth round bottom flask in a “V” geometry. The ends of two electrodes were submerged under 400 mL of anhydrous ethanol. The DC arc plasma was triggered by a welding machine (Panasonic 200BL3) between the electrodes. The arc current was fixed at 5 A for 30 min. During the whole process of the arc discharge, the flask was cooled by an ice water bath, and nitrogen was injected into the flask to ensure safety. The obtained solution was then centrifuged at 10,000 rpm for 5 min to remove suspended carbon impurities. 

SWCNTs prepared by enhanced direct injection pyrolysis synthesis [29] with an average diameter of around 0.9 nm were used as nanoreactors. The SWCNTs were first opened by thermal treatment in air at 450 °C for 30 min, and then the opened SWCNTs were immersed in a polyyne solution at 4 °C for 2 weeks to let polyynes enter the SWCNTs spontaneously. (6,5) SWCNTs were sorted by the aqueous two-phase technique using DNA. The details can be found in a previous study [30]. The (6,5) SWCNTs were already opened during the sorting, and they were deposited on a silicon substrate by drop casting and filled with polyynes utilizing the same process mentioned above. The filled samples were annealed in a dynamic vacuum at different temperatures of 200–900 °C for 1 h to coalesce the filled polyynes into LCCs.

### 2.2. Characterization

Absorption spectra were recorded using a UV-5200PC (METASH) spectrophotometer with a wavelength range of 200–400 nm, a scanning wavelength increment of 1 nm, and a cumulative scanning time of 1 s for each wavelength. Anhydrous ethanol was employed for baseline calibration. 

Raman spectra were measured using TriVista 557 (Princeton Instruments) equipped with lasers with wavelengths of 473, 532, 561, and 632.8 nm for excitation. The laser power was set below 1 mW to exclude heating effects on the samples. The slit width was set at 50 μm, and an X50 objective (N.A. = 0.5) was used; thus, the spectral resolution was ∼1 cm^−1^. All the spectra were calibrated by a Rayleigh scattering line. Filled and coalesced samples were normalized to the G-band of SWCNTs for ease of comparison. SERS was performed by mixing the polyyne solution with an Ag nanorod colloidal solution on a quartz substrate. The measurements were carried out before the solution dried.

The polyyne-filled SWCNTs were dispersed in ethanol. A drop of the suspended solution was put on a grid and dried at ambient conditions. The sample was measured by a high-resolution transmission electron microscope (HRTEM, FEI Titan) with a Cs-corrector performed at 80 kV. Images were recorded with an exposure time of 1 s. The contrast profiles of the images were evaluated using ImageJ.

## 3. Results and Discussion

**Figure 1:**
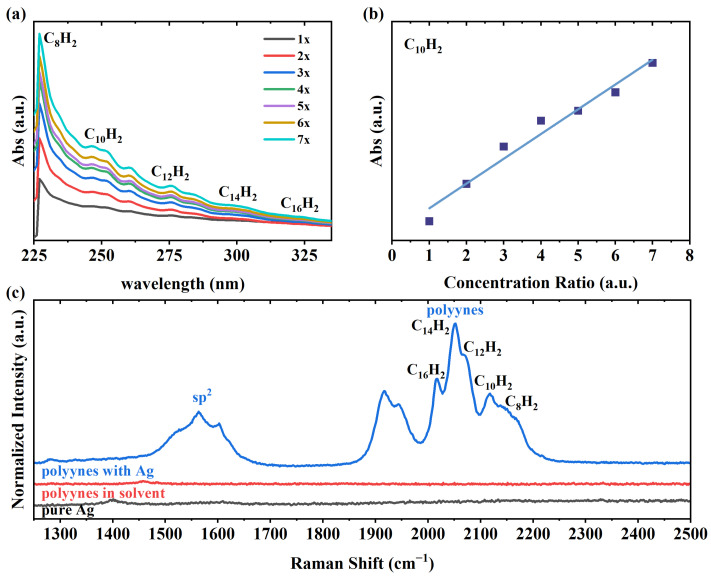
(**a**) Absorption spectra of the polyyne solution samples with increased relative concentrations. (**b**) The absorption intensity of C_10_H_2_ in (**a**) as a function of relative concentrations of the polyynes. (**c**) Raman spectra of the Ag solution, polyyne solution, and polyyne solution mixed with the Ag solution excited at 473 nm.

As depicted in Figure 1a, absorption spectra of the polyynes with increased concentrations consist of a series of signals that correspond to the polyynes with different lengths. By comparing the absorption spectra of the polyynes with certain lengths sorted by high-performance liquid chromatography, we can assign the signals to the polyynes with a certain length, i.e., C_2n_H_2_ (n = 4–8), in the sample [7]. By increasing the concentration of the polyynes in the ethanol solution, the absorption intensity of C_10_H_2_ increases gradually, following a linear relation, as shown in Figure 1b. Raman spectroscopy provides additional evidence of the existence of the polyynes. As shown in Figure 1c, no signal of polyynes can be seen when measuring the polyyne solution under non-resonance conditions since the optical energy gaps of those short polyynes are much greater than the energy of the laser. To enable observation of the polyynes, SERS was performed on the samples. The spectra of the Ag colloidal suspension did not exhibit any distinct characteristic band, whereas the Raman signals of the polyynes were significantly enhanced. As shown in Figure 1c, Raman bands of the polyynes are situated in the range of 1900 to 2250 cm^−1^. According to previous studies, the Raman signals of the polyynes can be recognized into two bands: the α-band and β-band at higher and lower frequencies, respectively [31]. In the SERS spectra, the bands generally exhibit downshifted frequencies compared to those conducted by conventional Raman spectroscopy; hence, they are denoted as the α′-band and β′-band. In our case, due to the presence of diverse polyynes, multiple peaks between 2000 and 2200 cm^−1^ are identified as the α′-band and are assigned to C_2n_H_2_ (n = 4–8) [31]. Since SERS spectra are contingent upon the nature and strength of the interaction of the adsorbed polyynes with the SERS enhancer and can also be influenced by the solvent, the bands exhibit a shift of approximately ten wavenumbers compared to the spectra of the polyynes prepared in alkanes using Au for SERS testing. In addition to the Raman bands of polyynes, the bands at around 1600 cm^−1^ are assigned to sp^2^ hybridized carbon [32,33] and are byproducts formed during the arc discharge between the graphite rods.

**Figure 2:**
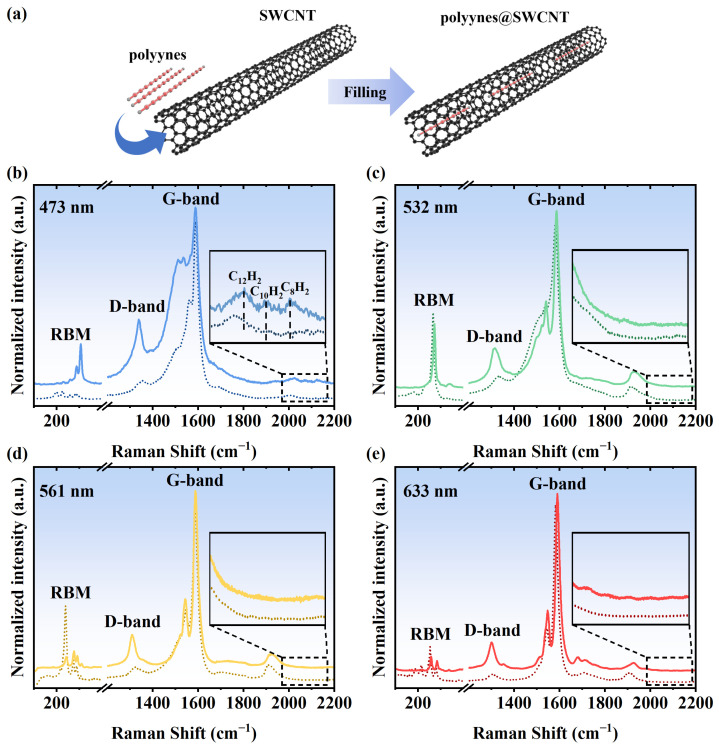
(**a**) Schematic diagram of the filling of polyynes inside an SWCNT. Raman spectra of the empty and polyyne-filled samples are plotted on dashed and solid lines, respectively, excited by lasers with wavelengths of (**b**) 473, (**c**) 532, (**d**) 561, and (**e**) 633 nm.

To increase the filling ratio of the polyynes, the average diameter of the SWCNTs used is slightly larger than that in previous studies [27]. Radial breathing mode (RBM) is a bond-stretching out-of-plane phonon mode with its frequency ω_RBM_ inversely related to the diameter (D) of SWCNTs according to the equation D = 234/(ω_RBM_ − 10) [34]. As shown in Figure 2, the RBM frequency is located in the range of 190–310 cm^−1^, suggesting that the diameter distribution is between 0.7 and 1.3 nm. Considering the main signals excited with different lasers are at around 270 cm^−1^, the average diameter is calculated to be around 0.9 nm, which is slightly larger than the previously used SWCNTs for filling polyyne with an average diameter ranging from 0.7 to 0.8 nm. Fundamentally, the larger diameter of the SWCNTs allows for a higher filling ratio of the polyynes, thereby facilitating the filling process executed at lower temperature to mitigate the risk of polyyne molecule decomposition compared to previous filling attempts conducted at 80 °C.

As shown in Figure 2, after filling the polyynes inside the SWCNTs, the RBM peaks shift to higher frequency due to the interaction between the SWCNTs and the polyynes [35,36]. Furthermore, the upshift of the G-band of the SWCNTs after filling indicates the charge transfer from the SWCNTs to the polyynes, suggesting p-doping by the polyynes. The intensity of the D-band of the SWCNTs increases extensively after the encapsulation of the polyynes. Considering the mild filling conditions, it is unlikely that the increase in the D-band can be attributed to the creation of defects on the walls of the SWCNTs but rather to the heterostructure of the polyynes@SWCNTs. All the above phenomena reveal that the polyynes were filled inside the SWCNTs. As shown in Figure 2b, a direct observation of the Raman signals from the filled polyynes was achieved without the use of SERS. This was possible due to the high filling ratio, which, along with the presence of SWCNTs enhanced the Raman signals of the polyynes. This is analogous to the increased signals observed for 6T molecules inside nanotubes [37]. The Raman modes of the polyynes are assigned to C_8_H_2_, C_10_H_2_, and C_12_H_2_, and their frequencies are consistent with previous reports for the polyynes inside thin SWCNTs [38]. Compared to the polyynes in the solution, the Raman peaks of the encapsulated polyynes are downshifted due to the charge transfer from the SWCNTs to the polyynes. Excited by other lasers with longer wavelengths than 473 nm, the signals of the polyynes are rather weak (Figure 2c–e) because the laser energies are far away from the resonance condition.

**Figure 3:**
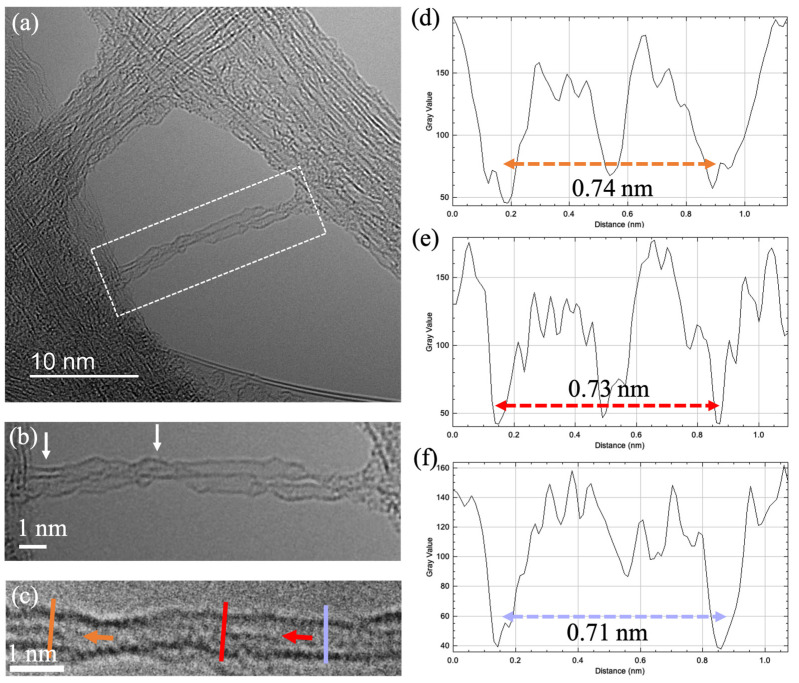
(**a**) An HRTEM image of overlapped SWCNTs. (**b**) The selected area in (**a**) is marked by a white dashed rectangle. (**c**) An HRTEM image of an individualized polyynes@SWCNT. The arrows indicate two polyynes. Contrast profiles of the polyynes@SWCNT across the (**d**) orange and (**e**) red lines. (**f**) Contrast profile of an empty SWCNT.

To further confirm the filling of the polyynes inside the SWCNTs, HRTEM characterizations were performed. As shown in Figure 3a, two short polyynes may exist in the same SWCNT, but it is also possible that the overlapping of the damaged SWCNTs is responsible for the false contrast. In order to clarify the encapsulation of polyynes inside an SWCNT, an individualized SWCNT is shown in Figure 3c. The diameter of the unfilled SWCNT is around 0.71 nm, which is slightly expanded when a polyyne is filled, as revealed by the contrast profiles in Figure 3d–f. The lengths of the two polyynes are around 1.21 and 1.48 nm, corresponding to the C_8_H_2_ and C_10_H_2_ polyynes, respectively.

**Figure 4:**
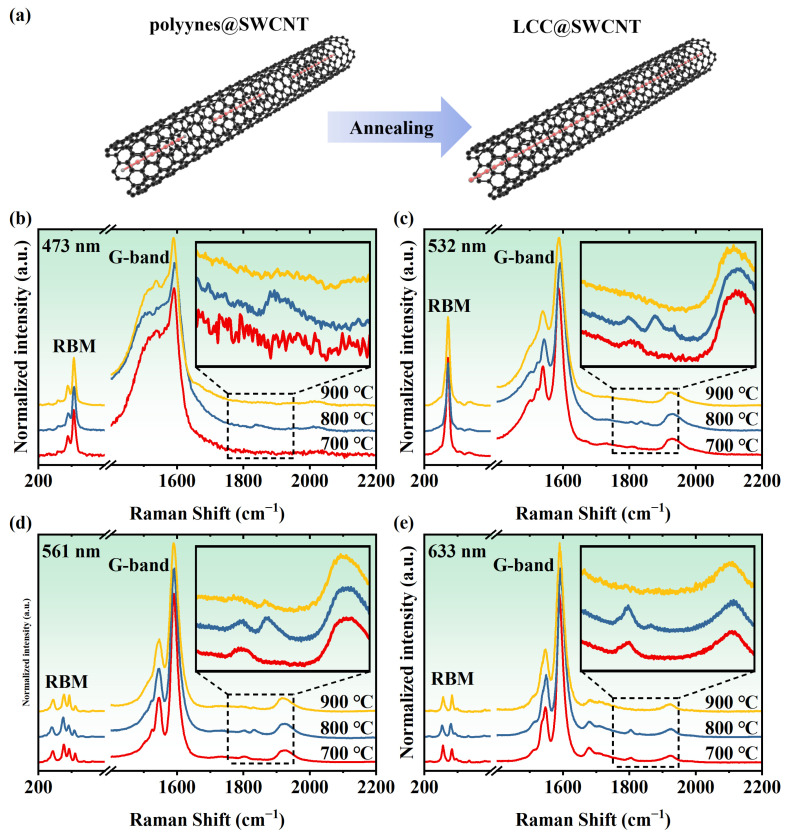
(**a**) Schematic diagram of coalescing the polyynes inside an SWCNT into an LCC by annealing. Raman spectra of the annealed polyyne-filled samples at different temperatures excited by lasers with wavelengths of (**b**) 473, (**c**) 532, (**d**) 561, and (**e**) 633 nm.

The polyynes@SWCNT samples were annealed at different temperatures to convert polyynes into LCCs (Figure 4a). It is crucial to carefully control the temperature during the coalescence process to ensure that the polyynes are effectively shielded from decomposition while allowing for the necessary coalescence between molecules. Several temperatures were tried for annealing. As shown in Figure 4b–e, Raman signals of polyynes disappear completely after annealing. Instead, new Raman signals located between 1800 and 1900 cm^−1^ show up, which reveals the formation of the LCCs from coalescence between polyynes. The optimal temperature for coalescence was found at 800 °C, which is much lower than the temperature at 1460 °C used in thermal annealing for the synthesis of LCCs inside DWCNTs without polyynes as precursors. The lower temperature at 700 °C was able to synthesize the LCCs with Raman signals at a lower frequency of around 1800 cm^−1^, which belongs to the long LCCs inside thinner tubes, as previously demonstrated [21,35]. Most of the LCCs were damaged when the samples were annealed at 900 °C.

**Figure 5:**
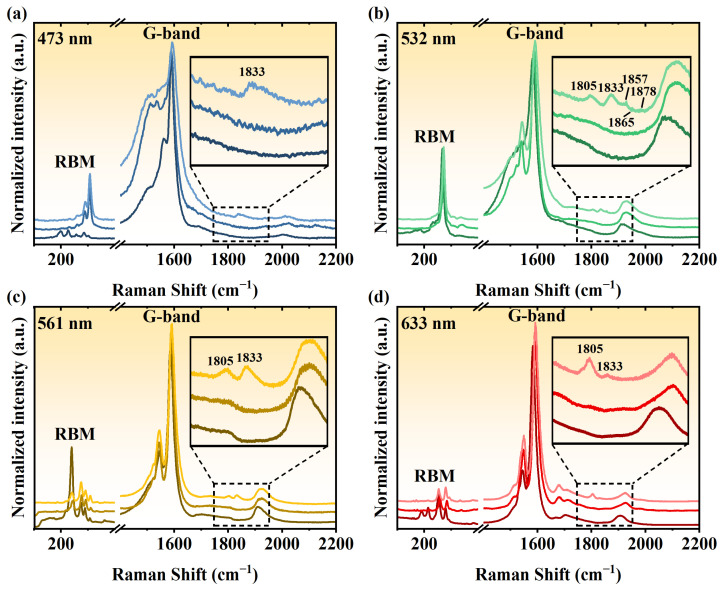
Raman spectra of empty, polyyne-filled, and polyyne-annealed SWCNTs from the bottom up excited by lasers with wavelengths of (**a**) 473, (**b**) 532, (**c**) 561, and (**d**) 633 nm.

To facilitate the analysis of the filling and annealing of the polyynes, Raman spectra of the optimized samples are plotted together, as shown in Figure 5. The disappeared Raman signals of the polyynes reveal the absence of the filled polyynes. In contrast, excited with different lasers, the LCCs inside various SWCNTs can be clearly observed. As shown in Figure 5, the variation in Raman frequencies between 1800 and 1900 cm^−1^ is attributed to the LCCs with different lengths and environmental interactions induced by different SWCNTs [21]. The peaks in the CC-band at 1805, 1833, and 1857 cm^−1^ referred to the LCCs with hundreds or thousands of carbon atoms, which is consistent with our previous observations for the LCC samples obtained from annealed DWCNTs [35]. In principle, LCCs consisting of tens of carbon atoms also exist in the sample, which has an intermediate length that is just in the transition range from polyyne to carbyne. According to the linear relation between Raman frequency and the optical energy gap of the LCC [35], a laser with energy 2.3–2.6 eV should be applied to excite those shorter carbon chains. With the help of a laser with a wavelength of 532 nm (2.33 eV), some weak signals up to 1900 cm^−1^ are indeed observed, as shown in Figure 5b. Unlike the long LCC, Raman frequencies of LCCs with intermediate lengths depend not only on the interaction with the hosted SWCNTs but also on their own lengths. Therefore, they are particularly important for studying the evolutionary process from polyyne to carbyne.

**Figure 6:**
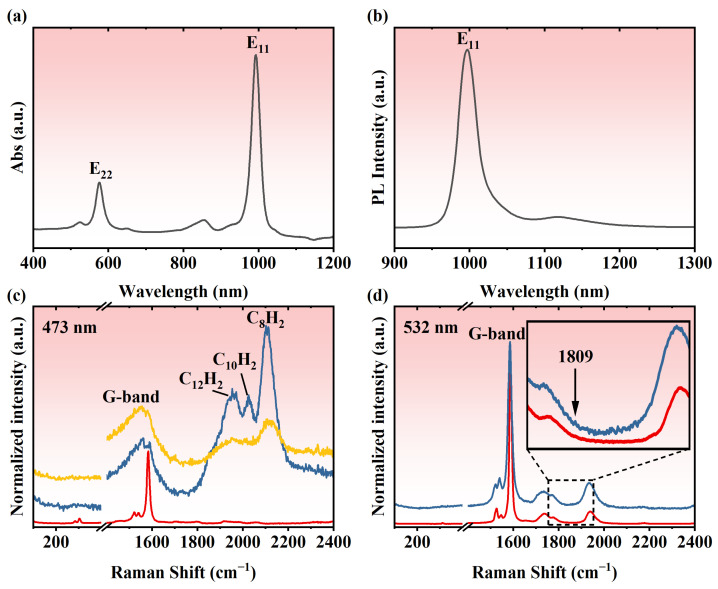
Absorption spectrum (**a**) and PL spectrum (**b**) of (6,5) SWCNTs. (**c**) Raman spectra of empty, polyyne-filled, and polyyne-annealed SWCNTs from the bottom up excited at 473 nm. (**d**) Raman spectra of polyyne-filled (**bottom**) and polyyne-annealed (**up**) SWCNTs excited at 532 nm.

Finally, single-chirality (6,5) SWCNTs were used for encapsulation of polyynes. (6,5) SWCNTs were sorted by the aqueous two-phase technique. The absorption spectrum and photoluminescence (PL) spectrum in Figure 6a,b reveal the high purity of the (6,5) SWCNTs. The Raman band of the polyynes with varying lengths of filling in the (6,5) SWCNTs is significantly greater than the G-band of SWCNTs (Figure 6c), which is in stark contrast to the chirality-mixed SWCNTs (Figure 2). This indicates an ultra-high filling ratio of the polyynes inside the (6,5) SWCNTs. Additionally, the intense Raman band may also be ascribed to the off-resonance condition of the (6,5) SWCNTs. The signals of the encapsulated polyynes decreased with increasing the annealing temperature and completely disappeared at 800 °C, suggesting that all the polyynes were decomposed into amorphous carbon and/or transformed into LCCs. Only an extremely weak signal of the LCCs can be seen in Figure 6d because the (6,5) SWCNTs were also damaged after the annealing at 800 °C. Therefore, although the filling ratio of the polyynes was extremely high, it was still challenging to coalesce the filled polyynes into the LCCs in the (6,5) SWCNTs by a normal annealing process. Fast heating using laser annealing may be an alternative way to overcome this challenge, which has been applied previously in our group for the synthesis of LCCs inside DWCNTs. Laser annealing is able to heat up the samples in seconds, allowing them to not only keep the integrity of the single-chirality SWCNTs but also coalesce the encapsulated polyynes into LCCs. This work is still ongoing.

## 4. Conclusions

By manipulating the diameter of the SWCNTs and filling conditions at low temperatures, we were able to achieve high-yield filling and subsequent coalescence of the polyynes inside SWCNTs, leading to the formation of LCCs. Importantly, Raman signals assigned to the LCCs with intermediate length were observed, which is the intermediate state of the transition from polyyne to carbyne. Particularly, (6,5) SWCNTs were also used for the synthesis of LCCs, which allows the properties of the LCCs to be tailored by the (6,5) SWCNTs. Moving forward, this research paves the way for further investigations into the controllable synthesis of LCCs with defined lengths, offering exciting prospects for exploring the SWCNT chirality-dependent physical properties of LCCs.

## Data Availability

The data presented in this study are available upon reasonable request from the corresponding author.
